# Glycomacropeptide Attenuates Inflammation, Pruritus, and Th2 Response Associated with Atopic Dermatitis Induced by 2,4-Dinitrochlorobenzene in Rat

**DOI:** 10.1155/2017/6935402

**Published:** 2017-02-07

**Authors:** Fabiola Carolina Muñoz, Maritza Montserrat Cervantes, Daniel Cervantes-García, Mariela Jiménez, Javier Ventura-Juárez, Eva Salinas

**Affiliations:** ^1^Department of Microbiology, Basic Science Center, Autonomous University of Aguascalientes, Av. Universidad No. 940, 20131 Aguascalientes, AGS, Mexico; ^2^Department of Morphology, Basic Science Center, Autonomous University of Aguascalientes, Av. Universidad No. 940, 20131 Aguascalientes, AGS, Mexico

## Abstract

Atopic dermatitis (AD) is one of the most common skin diseases, whose incidence is increasing in industrialized countries. The epicutaneous application of a hapten, such as 2,4-dinitrochlorobenzene (DNCB), evokes an experimental murine AD-like reaction. Glycomacropeptide (GMP) is a dairy bioactive peptide derived from hydrolysis of *κ*-casein by chymosin action. It has anti-inflammatory, prebiotic, and immunomodulatory effects. The present study was aimed to investigate the effect of GMP administration on DNCB-induced AD in rats. The severity of inflammatory process, pruritus, production of cytokines, and total immunoglobulin E (IgE) content were measured, and the histopathological features were analyzed. GMP reduced the intensity of inflammatory process and edema of DNCB-induced dermatitis, with a significant decrease in eosinophils recruitment and mast cells hyperplasia. In addition GMP suppressed the serum levels of total IgE and IL-4, IL-5, and IL-13 expression in AD-lesions. Besides, the levels of IL-10 were significantly increased. Remarkably, GMP administration before AD-induction abolished pruritus in dermatitis-like reactions in the rats. Taken together, these results indicate that GMP has an inhibitory effect on AD by downregulating Th2 dominant immune response, suggesting GMP as a potential effective alternative therapy for the prevention and management of AD.

## 1. Introduction

Atopic dermatitis (AD) is a chronic and relapsing skin disease that is characterized by skin inflammation and pruritus. It is one of the most common skin diseases, affecting about 15–30% of children and 2–10% of adults worldwide, with an increasing prevalence rate in industrialized countries [1]. Although it is not a life-threatening disease, AD has a significant impact on patients' quality of life and on economy of health services. Besides, AD is often the first manifestation of allergic disease, as most patients with AD will further develop another atopic disorder, such as allergic rhinitis or asthma [2].

The precise etiology of AD is not yet determined, but one possibility is a deregulation of adaptive and innate immune response raised by environmental and genetic factors [3]. In AD patients, genetic conditions, external stimuli, or scratching episodes disrupt barrier skin that facilitates allergen penetration and activation of keratinocytes to produce thymic stromal lymphopoietin that triggers dendritic cells to induce a Th2-cell mediated response [4]. In the acute phase of disease, infiltrated CD4+ T cells in skin lesions predominantly secrete IL-4, IL-5, and IL-13. These Th2 cytokines orchestrate a skin inflammation characterized by eosinophil recruitment and mast cells hyperplasia. Besides, IL-4 induces immunoglobulin (Ig)E isotype switching in B cells, increasing serum IgE levels which is associated with the pathogenesis of the disease [5]. In the chronic phase of the AD, Th1 cells appear and secrete interferon-gamma (IFN-*γ*) that is mainly associated with epidermal hyperplasia [1]. Therefore, the imbalance in the rate of Th1 and Th2 cells, or in Treg cells that maintain immune homeostasis locally, has special consideration in AD [6].

Animal models for human diseases are very important to analyze the mechanisms involved in the onset and development of pathologies and to establish treatment strategies for the disease [7]. Mice model has been widely used for the detailed study of AD and for the development of rapid trials of possible therapies for the disease [8]. Dermatitis model induced by skin repeated application of haptens causes histopathological, immunological, and clinical features similar to human AD [7, 9]. Although most of AD-models by hapten repeated application are developed in mice, thickness of the cornea layer and chemical permeability of skin in mouse are greater than rat and human, so rat skin suffers AD-like injuries less severe than mouse and more similar to human [10–12].

Many kinds of bioactive peptides that might prevent lifestyle-related diseases are released from food proteins after enzymatic digestion. Glycomacropeptide (GMP) is an active biopeptide derived from milk *κ*-casein that is released to the whey during cheese-making process by the action of chymosin [13]. It is composed of 64 amino acids extensively glycosylated with units of N-acetylneuraminic (sialic) acid that confers several nutraceutical and biological properties [14]. GMP has an excellent safe record and is not immunogenic [15]. As component of the whey, it is included in infant food formulas as a source of amino acids; besides, it is added to nutritional formulas for phenylketonuria patients due to the lack of phenylalanine [16]. Recently, GMP has deserved much interest for its proposed prebiotic, anti-inflammatory, and immunoregulatory properties. It has anti-inflammatory activity in rat models of colitis and ileitis induced by trinitrobenzene-sulphonic acid [17–19] and prevents extensive damage in colon in a model of colonic damage induced by dimethyl hydrazine [20]. Both GMP effects are mediated by the regulation of lymphocytes differentiation. Recent studies carried out in our laboratory show the prophylactic effect of orally administered GMP on the development of immune response associated with allergic sensitization, protecting animals from the severity of urticarial reaction and systemic anaphylaxis induced by allergens. This effect is related to changes in gut microbiota composition, upregulation of TGF-*β* and downregulation of IL-13 production by splenocytes, reduction in allergen-specific IgE production, and mast cells inhibition [21, 22]. GMP has also immunoregulatory activity in allergic asthma models, as it effectively suppresses blood and lung eosinophilia, goblet cell hyperplasia, and collagen deposition in airways. Beneficial effect of GMP in asthma is associated with downregulation of IL-5 and IL-13 and upregulation of IL-10 expression in asthmatic lung tissue [23].

The aim of this study was to evaluate whether oral GMP administration, previously or once pathology was established, can influence the development of AD. Firstly, we characterized a rat model of dermatitis by systemic sensitization followed by hapten repeated application. We further examined the effect of GMP in skin inflammation, pruritus, as well as Th2-immune response associated with AD to determine its potential prophylactic and therapeutic activity.

## 2. Material and Methods

### 2.1. Animals

Male Wistar rats (150–180 g) obtained from the Laboratory Animal Service of the Autonomous University of Aguascalientes were used throughout the study. Rats were housed under controlled conditions of temperature (22–24°C) and illumination (12 h light cycle) and fed with Rodent Laboratory Chow 5001 (Purina, Mexico City, Mexico) and tap water ad libitum. All experiments were carried out with strict adherence to ethical guidelines approved by the Institutional Normative Welfare Standards.

### 2.2. Protocol for Induction of Experimental Atopic Dermatitis

**Figure 1:**
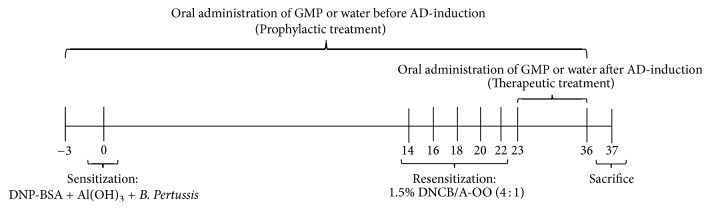
Schematic diagram of experimental dermatitis induction protocol and GMP administration. Rats were sensitized on day 0 with injection of DNP-BSA mixed with Al(OH)_3_ gel and simultaneously with* B. pertussis* vaccine. Animals were resensitized with topical application of DNCB in A-OO on days 14, 16, 18, 20, 22, and 36. Control group was injected with the adjuvants but without DNP-BSA and applied topically with A-OO mixture. GMP or water was administered, daily and orally, from 3 days before AD-induction or from day 23 after AD-induction, and until day 36 to analyze the prophylactic or therapeutic effect, respectively. Animals were sacrificed at day 37.

Ear cutaneous reaction was induced by repeated applications of 2,4-dinitrochlorobenzene (DNCB; Sigma, St. Louis, MO, USA) after systemic sensitization, as previously described [24]. Briefly, animals were sensitized at day 0, with an intramuscular injection of 1 mg of dinitrophenyl-bovine serum albumin (DNP-BSA) precipitated in 7.8 mg of aluminum hydroxide gel (Al(OH)_3_; Thermo Scientific, Waltham, MA, USA) in 1 mL of saline solution. Simultaneously, and as an adjuvant, 0.5 mL of* Bordetella pertussis* vaccine (Zuvirac, Mexico City, Mexico) containing 10–15 × 10^9^ heat-killed bacilli/mL was injected subcutaneously. On days 14, 16, 18, 20, 22, and 36, animals were resensitized with a topical application of 60 *µ*L of 1.5% w/v DNCB prepared in acetone-olive oil (A-OO) solution (4 : 1) to both sides of the right ear lobe of the rats. Control group was only injected with adjuvants and topically applied with A-OO solution (Figure 1).

### 2.3. Experimental Design

For characterization of dermatitis model, rats were randomly assigned to two different groups (5 rats per group): control and DNCB sensitized. For analysis of GMP effect, rats were randomly assigned to five different groups (8 rats per group): control, not sensitized and water administered before AD-induction; DNCB-P, DNCB sensitized and water administered before AD-induction; GMP-P, DNCB sensitized and GMP administered before AD-induction; DNCB-T, DNCB sensitized and water administered after AD-induction; and GMP-T, DNCB sensitized and GMP administered after AD-induction. GMP (Lacprodan® cGMP-10; a gift from Arla Foods Amba, Viby, Denmark) was orally administered to animals at 500 mg/kg/day dissolved in tap water. Oral intake of GMP was started from 3 days before sensitization to day 36 as prophylaxis (GMP-P) and from day 23 to day 36 when employed in a therapeutic manner, that is, once AD was established (GMP-T). Control, DNCB-P, and DNCB-T groups were administered orally with tap water during corresponding times (Figure 1). An esophageal catheter was used to deliver GMP solution or water. All animals were sacrificed with an overdose of ether at day 37, and blood and ear samples were obtained.

### 2.4. Evaluation of Ear Cutaneous Inflammatory Reaction and Edema

Cutaneous reaction was evaluated by ear swelling induced by the challenge with DNCB. Ear thickness was measured using a dial thickness gauge (Milomex, Ltd., Bedfordshire, UK) at 0, 1, 6, and 24 h after DNCB application on day 36. Ear swelling was calculated based in the increase of ear thickness as RT-LT, where RT and LT represent the thickness of the right and left ear, respectively, at the corresponding time point. At day 37 animals were sacrificed, the ears were excised from the base, and identical portions of the middle of the ears were removed using a metallic punch. The tissue samples were individually weighted on an analytical balance (Precisa XT220A, Dietikon, Switzerland). Edema was calculated based on the increase of ear weight as RW-LW, where RW and LW represent the weight of the fragment of the right and left ear, respectively.

### 2.5. Evaluation of Scratching Behavior

The total number of scratching events was counted during 10 minutes immediately after the application of DNCB on days 16, 22, and 36. For that purpose, rats were placed into an acrylic cage divided into eight compartments. Their behavior was recorded using a digital video camera (Samsung HMX-W350, New Jersey, USA). Videos were watched by two observers and the number of scratching events was counted. One scratching event or episode was defined as a series of one or more scratching movements by the hind paw directed toward the application site and ended when the rat either licked its hind paw or placed it back on the floor [25].

### 2.6. Histological Analysis

Upper portions of the right ears of each rat were fixed in 10% neutral formalin, embedded in paraffin, and sectioned into 5 *µ*m slices. Slices were stained with hematoxylin and eosin for evaluation of eosinophils infiltration and with toluidine blue for evaluation of mast cells number. After microscopic fields were photographed, the numbers of stained eosinophils and mast cells were counted in random areas (40,000 *µ*m^2^) with an AxioPlan Carl Zeiss microscope (Oberkochen, Germany) at 400x magnification. Three slides were stained per rat and three fields were examined per slide. Morphometric assessment was performed using AxioVision Rel 4.8 software by two observers who were not aware of the group of rats from which the samples originated.

### 2.7. Determination of Total IgE

Serum samples prepared from blood obtained on day 37 were stored at −70°C until used to IgE determination. Total IgE level in serum was quantified using a rat IgE ELISA kit (Abcam, Cambridge, UK) according to the manufacturer's instructions.

### 2.8. RNA Purification and Semiquantitative or Real-Time Quantitative PCR (qRT-PCR)

Total RNA was isolated from the lower ear tissue using the SV Total RNA Isolation System (Promega, Madison, WI, USA). Purified RNA was quantified with a NanoDrop 2000 Spectrophotometer (Thermo Scientific) with the A260/280 ratio. Only samples with ratio >1.8 were employed for cDNA synthesis. Reverse transcriptions of 2 *µ*g of RNA were performed with the RETROscript® Reverse Transcription kit (Thermo Scientific). Semiquantitative PCR was performed with 1 *μ*L of 1 : 10 diluted cDNA product, 5 *μ*L of PCR Master Mix 2x (Thermo Scientific), and 1 *μ*L of forward and reverse primers at 5 *μ*M each (listed on Table 1); all reactions were completed with nuclease-free water to 10 *μ*L. PCR conditions were as follows: initial denaturing at 95°C for 3 min, with 25, 30, or 35 cycles of 95°C for 30 sec, 60°C for 30 sec, and 72°C for 10 sec, and later for all reactions a final extension of 72°C for 3 min was included. Amplicons were separated in 2% agarose gels containing GelRed™ Nucleic Acid Gel Stain (Biotium, Hayward, CA, USA) as recommended by the manufacturer, in TBE 1x (89 mM Tris, 89 mM boric acid, 2 mM EDTA, pH 8). Gels were visualized under UV light in a MiniBis Pro documentation system (DNR Bio-Imaging Systems, Jerusalem, ISR). For RT-PCR, 2 *µ*L of diluted cDNA reaction was used as template for the detection of IL-4, IL-5, IL-13, IL-10, and *β*-actin with the GoTaq® qPCR Master Mix (Promega) in an Eco Real-Time PCR System (Illumina, San Diego, CA, USA). Relative quantification was determined with ΔΔCt method using *β*-actin as housekeeping gene for normalization.

### 2.9. Data Analysis

Data were expressed as mean ± standard error of the mean (SEM). Statistical analysis was performed by Student's *t*-test. Ear thickness data were analyzed by multicomparative Bonferroni test. Significance was set at *p* < 0.05.

## 3. Results 

### 3.1. Characterization of Dermatitis Evoked by Repeated Challenges with DNCB after Systemic Sensitization

**Figure 2:**
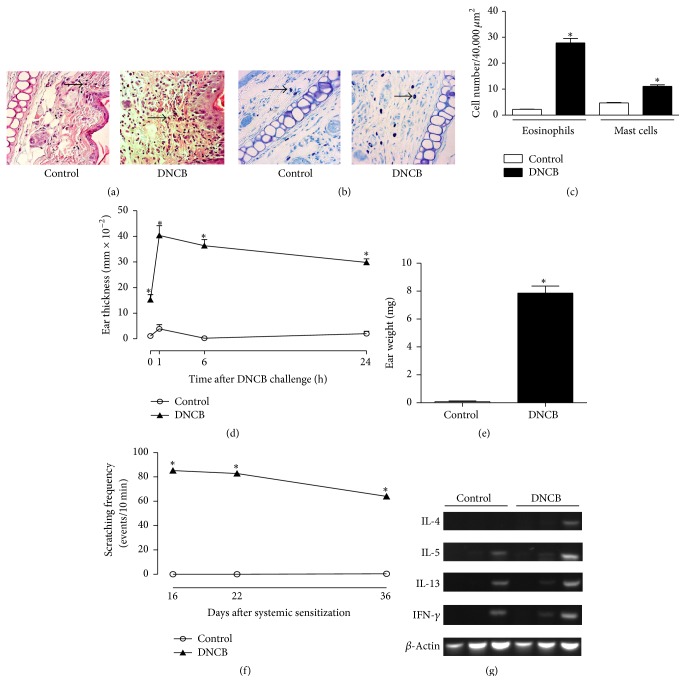
Characteristics of dermatitis-like reaction in rats challenged with DNCB after systemic sensitization. Histopathological features of the ears of control and DNCB challenged rats, 24 h after the sixth challenge, stained with (a) hematoxylin and eosin and (b) toluidine blue. Arrows indicated (a) eosinophils and (b) mast cells. (c) Eosinophils and mast cells were counted in dermis with a microscope at a magnification of 400x. (d) Ear thickness was measured at 0, 1, 6, and 24 h after last DNCB challenge. (e) To measure ear edema, equal areas from ears were punched and weighed 24 h after last challenge. (f) Scratching frequency was measured during the first 10 min after DNCB application and reported at days 16, 22, and 36. (g) Inflammatory cytokine mRNA expression in the skin lesion 24 h after the last DNCB challenge. Values represent mean ± SEM; *N* = 5 rats. ^*∗*^*p* < 0.001 versus control at each time point.

First, rats were systemically sensitized with DNP-BSA and later challenged 6 times by painting the right ear with DNCB/A-OO solution. As shown in Figures 2(a)-2(b), repeated impregnation with DNCB solution caused potent inflammatory changes in the ear skin, such as the thickening of both dermis and epidermis, edema, and the accumulation of eosinophils and mast cells. The number of eosinophils and mast cells in dermis of rats from DNCB group increased by 12.6- and 2.3-fold (Figure 2(c)). The ear thickness, measured as an indicator of skin inflammation [11], increased after each application of DNCB. On day 36, the ear thickness picket at 1 h after DNCB painting and maintained significantly greater than control rats at 6 and 24 h (Figure 2(d)). On day 37, edema in DNCB group was 98-fold higher than that in control rats (Figure 2(e)). Scratching toward the ear receiving DNCB application was observed from day 16. Scratching occurred immediately after the application of DNCB, with its frequency decreasing as time passed, and no scratching was observed at 1 h and thereafter. The scratching events counted for the first 10 min, as shown in Figure 2(f), significantly increased at day 16 and were almost equal at day 22, with a slight decrease at day 36. Total RNA was extracted from the skin lesions excised 24 h after the sixth DNCB challenge and the expression of inflammatory cytokines was examined. As shown in Figure 2(g), the IFN-*γ*, IL-5, and IL-13 mRNA expression in skin of control rats was very weak, but it was potentiated in DNCB group. Furthermore, although the expression of IL-4 mRNAs in skin was undetectable in control rats, DNCB-treatment induced their expression in dermatitis lesion.

### 3.2. Oral GMP Administration Diminishes Inflammatory Process in Dermatitis

**Figure 3:**
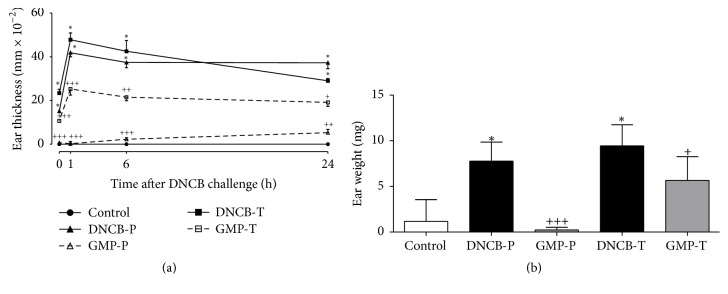
Effect of GMP administration on inflammatory process. (a) Ear thickness value is represented by the difference between right and left ear, at day 36. (b) Edema is represented by the difference between right ear weight and left ear weight at day 37. Data are presented as mean ± SEM, *N* = 8. Control, not sensitized and water administered before AD-induction; DNCB-P, DNCB sensitized and water administered before AD-induction; GMP-P, DNCB sensitized and GMP administered before AD-induction; DNCB-T, DNCB sensitized and water administered after AD-induction; and GMP-T, DNCB sensitized and GMP administered after AD-induction; ^*∗*^*p* < 0.0001 versus control; ^+^*p* < 0.02; ^++^*p* < 0.002; ^+++^*p* < 0.0001 versus the respective DNCB without GMP administration at each time point.

First we investigated whether oral intake of GMP might modify the development of the inflammatory response associated with dermatitis. So, ear thickness was measured after DNCB-repeated applications. On day 36, before the sixth DNCB application (0 h), DNCB-P and DNCB-T animals reported an increase of 0.15 and 0.23 mm over control animals. But animals administered with GMP reduced in 95.6 and 54.55% the thickness induced by the previous five DNCB applications when used in a prophylactic or therapeutic manner, respectively. One hour after the last DNCB application, ear thickness presented a peak of 0.41 and 0.48 mm in the ears of DNCB-P and DNCB-T animals, which was sustained at 6 h and presented a slight decrease at 24 h. However, when animals were GMP administered before AD-induction the inflammatory process was reduced in 99.4, 93.98, and 85.89% at 1, 6, and 24 h after challenge, and if they received GMP after AD-induction the ear inflammation was diminished in 47.16, 49.41, and 34.06% (Figure 3(a)).

Another way to assess changes in the inflammatory process is to evaluate the ear edema as the increment in ear weight. As shown in Figure 3(b), when animals were repeatedly challenged with DNCB the ear edema was 6.64 (DNCB-P) and 8.05 (DNCB-T) higher than in control animals. However, when animals were GMP administered before AD-induction there was a decrease of 97.03% on ear DNCB-induced edema. Besides, animals that received GMP once dermatitis was established showed a decrease of 39.87% on edema when compared to untreated group (DNCB-T).

### 3.3. Scratching Behavior Is Inhibited by GMP-Prophylaxis

**Figure 4:**
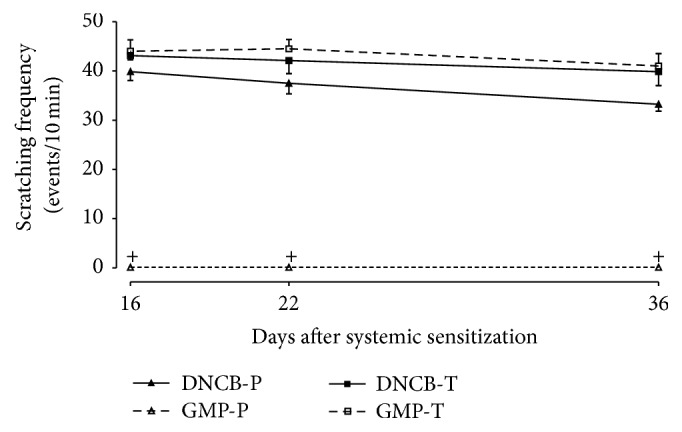
Effect of GMP administration on scratching frequency. Events of scratching were measured after DNCB challenge during 10 minutes. Data are presented as mean ± SEM, *N* = 8. DNCB-P, DNCB sensitized and water administered before AD-induction; GMP-P, DNCB sensitized and GMP administered before AD-induction; DNCB-T, DNCB sensitized and water administered after AD-induction; and GMP-T, DNCB sensitized and GMP administered after AD-induction; ^+^*p* < 0.0001 versus DNCB-P at each time point. GMP-T versus DNCB-T was ns.

Pruritus is one of the major symptoms of AD and impacts quality of life of patients in a significant manner [26]. Control animals did not show any scratching event in the right ear during 10 min immediately after the application of A-OO mixture (data not shown). The chronological profile of scratching behavior in DNCB challenged rats, treated or not with GMP, is shown in Figure 4. In DNCB-P and DNCB-T rats the number of scratching events remained almost constant during 10 min after DNCB topical application at days 16, 22, and 36, with an average of 36.87 and 41.71 scratching events. Oral GMP administration before AD-induction resulted in a significant and dramatic inhibition of more than 99% in the number of scratching episodes of DNCB-applied animals during the same days, pruritus being almost completely abolished. In contrast, there were no differences in scratching behavior between GMP-T and DNCB-T groups, indicating that GMP has no effect on pruritus when it was administered once dermatitis was established.

### 3.4. GMP Administration Reduces the Infiltration of Inflammatory Cells into DNCB-Induced Skin Lesions

**Figure 5:**
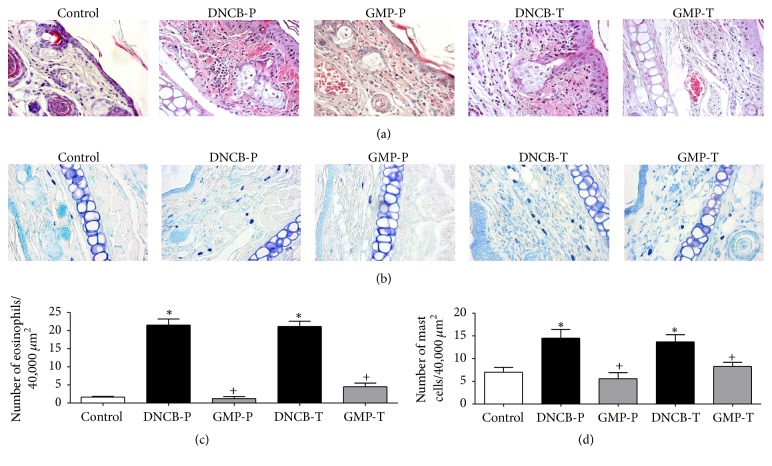
Effect of GMP on inflammatory cell infiltration. Sections of right ears were stained with (a) hematoxylin and eosin to identify eosinophils and (b) blue toluidine for mast cells. Quantitative analysis of (c) eosinophils and (d) mast cells per 40,000 *μ*m^2^ of dermis was developed with a microscope at magnification of 400x. Data are presented as mean ± SEM, *N* = 8. Control, not sensitized and water administered before AD-induction; DNCB-P, DNCB sensitized and water administered before AD-induction; GMP-P, DNCB sensitized and GMP administered before AD-induction; DNCB-T, DNCB sensitized and water administered after AD-induction; and GMP-T, DNCB sensitized and GMP administered after AD-induction; ^*∗*^*p* < 0.0001 versus control; ^+^*p* < 0.0001 versus the respective DNCB without GMP administration.

Cellular changes in dermatitis skin include marked infiltration of eosinophils and mast cells hyperplasia [27]. Histological analysis revealed that topical DNCB elicited the infiltration of inflammatory cells into ear skin lesion but GMP administration attenuated the amount of infiltrated inflammatory cells (Figures 5(a)-5(b)). Morphometric assessment showed that the number of eosinophils in ears with DNCB applications was 13.12 and 12.89 times higher than in control group, for DNCB-P and DNCB-T rats. Whereas in animals administrated with GMP, amount of eosinophils in dermis was reduced in 94.47% when GMP was used as prophylaxis or 78.71% when it was administered in a therapeutic manner (Figure 5(c)). On the other hand, the amount of mast cells in the dermis of DNCB untreated animals was 2-fold compared to control animals. However, mast cells number was remarkably lowered in 61.51% by GMP administration before AD-induction and in 39.59% by GMP administration after AD-induction (Figure 5(d)).

### 3.5. Influence of GMP on Serum Levels of IgE

**Figure 6:**
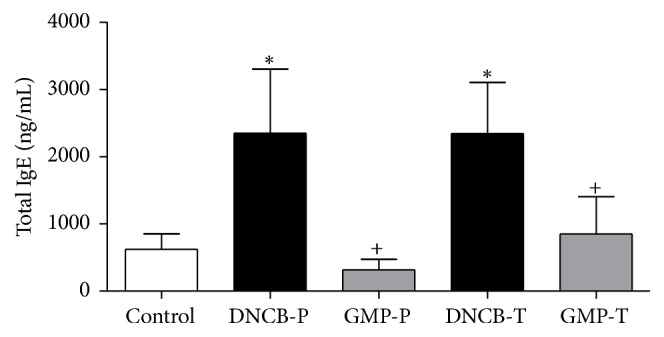
Effect of GMP on total serum IgE. Serum was collected 24 h after last challenge with DNCB. IgE level was measured by ELISA. Data are presented as mean ± SEM, *N* = 8; control, not sensitized and water administered before AD-induction; DNCB-P, DNCB sensitized and water administered before AD-induction; GMP-P, DNCB sensitized and GMP administered before AD-induction; DNCB-T, DNCB sensitized and water administered after AD-induction; and GMP-T, DNCB sensitized and GMP administered after AD-induction; ^*∗*^*p* < 0.001 versus control; ^+^*p* < 0.001 versus the respective DNCB without GMP administration.

It is known that dermatitis is characterized by high levels of serum total IgE [28]. Therefore, we investigated whether GMP suppresses IgE in serum. After the sixth DNCB application, serum samples were collected and total IgE levels were measured by ELISA. In rats receiving topical DNCB total IgE levels were 3.7-fold higher than in control group. Prophylaxis with GMP significantly reduced in 86.53% total serum IgE levels as compared with nontreated animals (DNCB-P). When GMP was administered in a therapeutic manner the decrease in IgE level was 63.68% (Figure 6).

### 3.6. Effect of GMP Administration on IL-4, IL-5, IL-13, and IL-10 Expression in Dermatitis Skin Lesion

**Figure 7:**
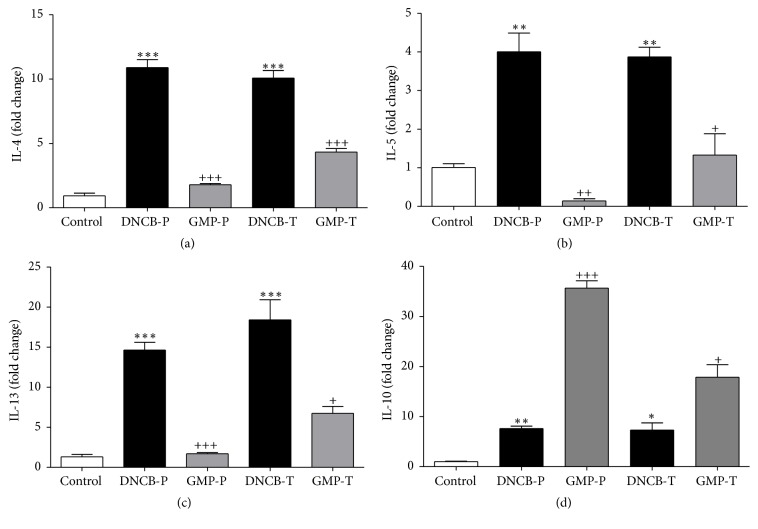
Effect of GMP on mRNA expression for IL-4, IL-5, IL-13, and IL-10 in ear tissue. Changes in (a) IL-4, (b) IL-5, (c) IL-13, and (d) IL-10 mRNA expression relative to *β*-actin were measured. Skin tissue was obtained at day 37 from control and DNCB sensitized rats, administered or not with GMP. Three rats from each experimental group were analyzed. Skin samples from each rat were analyzed in triplicate for qPCR. Each value represents the mean ± SE. Control, not sensitized and water administered before AD-induction; DNCB-P, DNCB sensitized and water administered before AD-induction; GMP-P, DNCB sensitized and GMP administered before AD-induction; DNCB-T, DNCB sensitized and water administered after AD-induction; and GMP-T, DNCB sensitized and GMP administered after AD-induction; ^*∗*^*p* < 0.05, ^*∗∗*^*p* < 0.001, and ^*∗∗∗*^*p* < 0.0001 versus control; ^+^*p* < 0.05, ^++^*p* < 0.01, and ^+++^*p* < 0.0001 versus the respective DNCB without GMP administration.

Inflammation in AD is mediated by an initial Th2 phase, which is orchestrated by IL-4, IL-5, and IL-13 cytokines and is related to IgE production and eosinophilia [1]. To address the question whether GMP administration might modulate this Th2 inflammatory response in dermatitis, we examined mRNA changes of IL-4, IL-5, and IL-13 by qRT-PCR in injured skin tissue. We found that IL-4, IL-5, and IL-13 mRNA were 11.24-, 3.93-, and 12.50-fold higher, on average, in DNCB groups. Interestingly, GMP administration before AD-induction decreased in 83.56, 96.5, and 88.38% the expression of IL-4, IL-5, and IL-13 in dermatitis skin. When GMP was administered after AD-induction the decrease of these Th2-inflammatory cytokines, although not so marked, was still significant in order of 57.05, 65.89, and 63.3% lower than nontreated animals (Figures 7(a)–7(c)).

Besides, we analyzed mRNA changes on IL-10, one of the most important anti-inflammatory cytokines which downregulates the immune system minimizing tissue damage during inflammation [29]. As shown in Figure 7(d), the expression of IL-10 was significantly higher in DNCB challenged than in control animals, but it was clearly potentiated by GMP administration. IL-10 expression was 4.68-fold higher in DNCB challenged animals when receiving GMP as prophylaxis and 2.44-fold higher when it was administered in a therapeutic manner.

## 4. Discussion

AD is one of the most common skin inflammatory disorders [30] and its early onset in childhood often triggers the atopic march, which leads to the consequent development of asthma and allergic rhinitis [31]. The anti-inflammatory therapy of AD includes topical corticosteroids and calcineurin inhibitors; however resolution is often temporary and long-term usage can be associated with significant adverse effects [32, 33]. Due to the deleterious effect of AD on the quality of life of patients and the significant economic impact in health systems, new therapies that prevent or act on the immunological mechanisms involved in AD and with minimal side effects are required.

In this study we investigated whether GMP attenuates the severity of AD-like lesions induced by DNCB in rat. We chose rat as animal model because thickness of the corneal layer and chemical permeability of skin rat is more similar to human than mice [10–12]. So, we firstly characterized an experimental model of AD in rat based on a reported protocol of epicutaneous DNCB sensitization in mice [24]. The rat model demonstrates immunological dysregulation, such as IgE hyperproduction in serum and elevated IL-4, IL-5, IL-13, and IFN-*γ* expression in skin injuries. It also shows hypertrophy of epidermis, intracellular edema, and infiltration of inflammatory cells, such as eosinophils and mast cells, which are histopathological features of AD [1]. Besides, DNCB applications induce a scratching behavior toward the affected area that denotes the appearance of pruritus, one of the most characteristic AD symptoms [26]. So, in our rat model AD-like lesions have histopathological, immunological, and clinical features of human lesions.

GMP is a bioactive peptide that has been demonstrated to prevent allergic sensitization and attenuate the severity of urticarial reaction, anaphylaxis, and asthmatic airway inflammation and remodeling [21, 23]. It is already incorporated in nutritional products and is safe and not immunogenic [15–17]. In the present study, we demonstrated that oral administration of GMP in a prophylactic or therapeutic manner induces a significant reduction in the development of AD by strongly reducing skin inflammation, eosinophils, and mast cells number in dermis and total IgE levels. Besides, GMP administration targets the Th2-inflammatory response, as it decreases IL-4, IL-5, and IL-13 but increases IL-10 expression in AD-like skin lesions. Prophylaxis with GMP also impacts on pruritus, as it suppresses scratching episodes associated with disease. So, we demonstrate that oral intake of GMP before or after AD establishment modulates immune response and pathophysiology in experimental AD.

The epidermis of AD patients is characterized by significant skin barrier disruption which activates keratinocytes to develop an extreme Th2-dominant response that strengthens IgE production [34]. Thus, IgE level in the serum is correlated with the severity of AD [5]. In our experimental model of AD, high levels of total IgE were quantified in serum. GMP administration before or after AD-induction significantly reduces serum total IgE levels. When GMP was administered before AD-induction IgE levels were lower than in control animals, suggesting that in this condition GMP administration can suppress serum total IgE. It is known that IgE released from B cells binds to mast cells. Allergens induce mast cells degranulation through IgE-Fc*ε*RI complex and the release of several biological mediators involved in skin inflammation [35]. So, a lessened level of IgE is in line with the reduction of edema and skin inflammation of AD-lesions observed in animals with GMP administration. Previously, it has been demonstrated that GMP inhibits mast cells activation by allergens [22] and we observed a reduced number of mast cells in dermis of GMP-treated animals, so the reduction in edema and skin inflammation as a consequence of GMP administration might also be mediated by alterations in mast cells number and function.

One of the central causes of the AD is the dysregulated Th1 and Th2 response that induces the characteristic Th2-dominant skin allergic inflammation [36]. In this Th2 response, the involvement of IL-4, IL-5, and IL-13 is crucial in humans [37]. In transgenic mice that overproduce IL-4, IL-5, and IL-13, investigators have demonstrated a positive correlation between the onset and progression of AD-like disease and the expression of these Th2 cytokines [38]. In our experimental model of AD the expression of IL-4, IL-5, and IL-13 was increased in skin lesions. It is known that IL-5 plays an important role in eosinophil differentiation, activation, proliferation, and chemotaxis [39, 40]. The number of eosinophils and levels of IL-5 have previously been shown to be elevated in injured skin of patients with AD [5, 41]. We show that GMP administration before or after AD-induction induces a significant reduction in IL-5 expression in AD-lesions, which is correlated with the decrease in the number of eosinophils infiltrated in dermis. On the other hand, transgenic mice overexpressing epidermal IL-4 or IL-13 spontaneously developed signs and symptoms associated with AD, including elevated IgE levels [42, 43]. So, reduced levels of IL-4 and IL-13 in skin of animals treated with GMP in a prophylactic or therapeutic manner are in concordance with the decrease in total IgE. The downregulation of the Th2-dominant skin inflammation by GMP administration may be associated with the increased expression of IL-10, a known regulatory cytokine. It has been reported that IL-10 inhibits both the proliferation and the cytokine synthesis of CD4+ Th2 cells [44]. Recently, the role of IL-10 in the control of AD development and maintenance has been highlighted by the fact that polymorphisms in the IL-10 gene could represent a genetic marker for AD in childhood [45]. As Th2 cytokines destabilize cutaneous barrier function [46, 47] and IFN-*γ* is crucial in dermal thickening and in the progression to chronic AD skin lesions [1], the study of the effect of GMP administration on the recovery of skin barrier integrity and on levels of IFN-*γ* expression is the aim of our current research.

Pruritus is a clinical manifestation of AD [26] that causes a great deterioration in patient's quality of life [48]. Besides, scratching worsens the dermatitis, increasing lesions in skin and thereby aggravating pruritus [49]. Thus, proper treatment of pruritus is the critical part of therapeutic approach to AD. Our rats with dermatitis showed an intense pruritus after DNCB application, but prophylaxis with GMP totally abolished the scratching episodes of the rats. A wide range of itch-inducing stimuli generated within the skin are able to trigger pruritus. Among them, histamine is recently considered relevant, as combined H1R/H4R antagonists therapy is successfully addressing pruritus in AD [50]. The decrease in IgE levels and mast cells number observed in animals administered with GMP before AD-induction, together with the reported inhibitory action of GMP on mast cells activation by allergen [22], might cause a decrease in histamine levels in skin, impacting on itching. Besides, it has been reported that transgenic mice expressing IL-13 in skin develops intense pruritus [43]. Dupilumab, a monoclonal antibody that binds to IL-4R*α* and blocks both IL-4 and IL-13 signaling pathways, induces a reduction in the pruritus score of patients with moderate to severe AD [51, 52]. These data indicate a role of IL-4 and IL-13 in triggering pruritus. Thus, antipruritic action of GMP-prophylaxis might be also mediated by the reduction of IL-4 and IL-13 expression in skin. However, due to the wide range of stimuli able to trigger pruritus in AD we cannot exclude a possible effect of GMP on other itching-inducing element.

GMP exerted a clearly superior therapeutic effect when it was given before AD-induction than when administered once AD-lesions were established. This is a common observation with GMP, because when it is used as anti-inflammatory therapy in experimental colitis its effect is greater when used as prophylaxis [17]. We recently demonstrated that GMP administration before allergen sensitization induces a significant increase in the amount of* Lactobacillus*,* Bifidobacterium*, and* Bacteroides *in the gut of sensitized animals [22]. In this regard, data about the effect of probiotics in the prevention and treatment of AD remain elusive, with negative and positive results, but evidencing that their positive effects depend on factors such as the type of probiotic strain, method of administration, onset time, duration of exposure, and dosage [53]. Particularly* Lactobacillus* and* Bifidobacterium* as therapy in AD show a promissory effect on prevention of pediatric AD, while there is less convincing information about their effects when used in a therapeutic manner [54], which is in concordance with our results. It is important to highlight that even after AD-induction most of the beneficial effects of GMP were retained, with exception of antipruritic effect. This may be due to the lesser reduction in IgE levels, mast cells number, and IL-4 and IL-13 expression in animals administered with GMP once AD was established. The remaining levels of these immune elements might be sufficient to maintain pruritus in the animals. However, we must consider that patients with AD may benefit from anti-inflammatory and Th2-downregulation properties of GMP used in a therapeutic manner.

In conclusion, the present study shows that GMP possesses prophylactic and therapeutic effects in the development of AD. GMP effectively suppresses skin inflammation, eosinophils recruitment, and mast cells hyperplasia in dermis, as well as total IgE in serum. Beneficial effect of GMP is associated with downregulation of IL-4, IL-5, and IL-13 expression together with upregulation of IL-10. Prophylactic administration of GMP also abolished pruritus. This study provides the first experimental basis for the potential use of GMP in the prevention and therapy of AD.

## Figures and Tables

**Table 1:** Oligonucleotides for gene expression quantification.

Gene	Oligonucleotides	Accession number
IL-4	Fw: CACCTTGCTGTCACCCTGTT	NM_201270.1
	Rv: ACATCTCGGTGCATGGAGTC	
IL-5	Fw: CAGTGGTGAAAGAGACCTTG	NM_021834.1
	Rv: GTATGTCTAGCCCCTGAAAG	
IL-13	Fw: ATCGAGGAGCTGAGCAACAT	NM_053828.1
	Rv: ATCCGAGGCCTTTTGGTTAC	
IFN-*γ*	Fw: GCCTAGAAAGTCTGAAGAAC	NM_138880.2
	Rv: GAGATAATCTGGCTCTCAAG	
IL-10	Fw: CACCTTGCTGTCACCCTGTT	NM_012854.2
	Rv: ACATCTCGGTGCATGGAGTC	
*β*-Actin	Fw: GTCGTACCACTGGCATTGTG	NM_031144.3
	Rv: GCTGTGGTGGTGAAGCTGTA	
